# The inoculation with* Pseudomonas simiae* WCS417 strain promotes growth and the induction of iron-deficiency responses in cucumber plants (*Cucumis sativus* L.)

**DOI:** 10.1007/s00425-025-04844-5

**Published:** 2025-10-29

**Authors:** Miguel A. Aparicio, Francisco J. Ruiz-Castilla, José Ramos, Francisco J. Romera, Carlos Lucena

**Affiliations:** 1 Departamento de Agronomía. Edificio Celestino Mutis (C-4), Campus de Excelencia Internacional Agroalimentario de Rabanales (ceiA3), University of Córdoba, 14014 Córdoba, Spain; 2 Departamento de Química Agrícola, Edafología y Microbiología. Edificio Severo Ochoa (C-6), Campus de Excelencia Internacional Agroalimentario de Rabanales (ceiA3), University of Córdoba, 14014 Córdoba, Spain

**Keywords:** Biofertilizer, Chlorosis, Cucumber, Ferric reductase, Hydroponics, Iron uptake, PGPR

## Abstract

**Main conclusion:**

Inoculation with *Pseudomonas simiae* WCS417 improves cucumber growth under Fe deficiency conditions and induces iron-deficiency responses, making it a promising candidate for sustainable biofertilization strategies in dicot plants.

**Abstract:**

Iron (Fe) deficiency poses a significant agronomic challenge in calcareous soils, particularly affecting dicot plants. Conventional production methods rely heavily on high-yielding varieties and the application of substantial amounts of agrochemicals, leading to considerable environmental concerns. In this context, leveraging the potential of beneficial rhizosphere microorganisms as biofertilizers represents a highly promising and environmentally sound alternative to chemical fertilizers. This study aims to investigate the efficacy of the nonpathogenic strain *Pseudomonas simiae* WCS417 in eliciting Fe deficiency responses in cucumber plants, along with its impacts on plant growth and Fe chlorosis. Conducted under hydroponic conditions, our experiments reveal compelling outcomes. Root inoculation of cucumber plants with *P. simiae* significantly enhances plant growth while concurrently mitigating Fe chlorosis symptoms over successive cultivation days. The inoculation with this bacterium induces acidification in the subapical zone of cucumber roots, facilitating Fe solubility in the rhizosphere. Additionally, *P. simiae* triggers the upregulation of Fe-related genes in inoculated plants, even under Fe sufficiency. In conclusion, *P. simiae* emerges as a potent enhancer of Fe deficiency responses in cucumber plants. Its ability to promote growth, enhance Fe solubility through rhizosphere acidification, and alleviate Fe chlorosis underscores its potential as an effective biofertilizer for a sustainable Fe nutrition of dicot plants.

## Introduction

Fe is an essential element for plant growth (Marschner 2012). This element is abundant in most soils but is present in chemical forms generally not available to plants. As a result, plants enhance acquisition of Fe through morphological and physiological responses in their roots, aimed at facilitating Fe mobilization and uptake (Ivanov et al. 2012; Kobayashi and Nishizawa 2012; Brumbarova et al. 2015; Lucena et al. 2015). Deficiency of this nutrient initiates root responses, such as development of subapical root hairs and the acidification of the rhizosphere or the production of organic acids (Lucena et al. 2015, 2018; Song and Liu 2015; Neumann 2016; Stetter et al. 2017; Bhosale et al. 2018). A defining feature of dicot plants (Strategy I plants) is the need to reduce Fe(III), the most abundant form of Fe in soils, to Fe(II), prior to acquisition. In *Arabidopsis*, this Fe(III) reduction is catalyzed by a ferric reductase (EC 1.16.1.17) encoded by the *FRO2* gene, whereas Fe(II) uptake is mediated by a transporter encoded by the *IRT1* gene (Walker and Connolly 2008; Ivanov et al. 2012). The acidification of the rhizosphere is due to upregulation of *AHA* or *HA* (H^+^-ATPase) genes (Waters et al. 2007; Brumbarova et al. 2015; Lucena et al. 2015). The acidification facilitates the solubilization of Fe and the functioning of the ferric reductase, which has an optimum pH around 5.0 (Lucena et al. 2007; Waters et al. 2007). All these genes are up-regulated under Fe deficiency and are activated by specific bHLH transcription factors, which are also upregulated by Fe deficiency. These bHLH transcription factors include FIT, bHLH38, bHLH39, and others in *Arabidopsis* (Yuan et al. 2008; Ivanov et al. 2012; Brumbarova et al. 2015).

For a long time, soil microorganisms have been a forgotten element. Modern agriculture alleviates nutritional deficiencies with the indiscriminate contribution of agrochemicals, while it mitigated the effect of pests and diseases with the use of phytosanitary products. Fortunately, that dynamic has been gradually changing. There are numerous studies that reveal the potential of the microorganisms present in the rhizosphere as an adjuvant in mineral nutrition (Masalha et al. 2000; De Santiago et al. 2019; Aparicio et al. 2023, 2025; Sevillano-Caño et al. 2024). Likewise, it is evident that this alternative could alleviate the negative effects of the agronomic techniques carried out during the last twentieth century, thus promoting a circular economy and facilitating greater profitability for the farmer (Allouzi et al. 2022). Having soil microorganisms as facilitators of mineral nutrition means facing problems of water eutrophication, runoff, leaching, soil degradation, loss of diversity, and greenhouse gas emissions derived from the use of chemical fertilizers (Torres and Capote 2004). Likewise, the excessive accumulation of heavy metals in water and food, as a consequence of the use of agrochemicals, has entered the food chain, putting both human health and that of other living beings at risk (Khan et al. 2018; Khatun et al. 2023). Consequently, this alternative focused on the rhizosphere is not only more respectful, but also adjusts to current government measures that aim to respond to the production and quality of crops, guaranteeing the protection of the environment and public well-being (Ryan et al. 2012). Therefore, achieving maximum knowledge of the microbial communities that inhabit our cultivated soils to achieve the greatest performance of the rhizospheric microbiome is crucial to enhance the agricultural sector, as already stated in numerous articles and reviews (Gray and Smith 2005; Merzaeva and Shirokikh 2006; Glick 2012; Shen et al. 2013; Bulgarelli et al. 2015; Edwards et al. 2015; Backer et al. 2018; Kumar and Dubey 2020).

The zone of soil in proximity to plant roots, known as the rhizosphere, spans from 2 to 80 mm from the root (Koo et al. 2005). This region hosts a diverse microbial community, reaching densities of up to 10^10^ bacteria per gram of soil (Gans et al. 2005; Roesch et al. 2008), comprising a wide array of taxa (Kyselková et al. 2009; Gomes et al. 2010). However, some studies indicate that microbial biodiversity in the rhizosphere does not exceed that of the surrounding bulk soil, despite the influence of root exudates (Berendsen et al. 2012). While prevailing knowledge of plant–microbe interactions has centered on the pathogenic impact of microorganisms on plants (Zilber-Rosenberg and Rosenberg 2008), the reality is that most microorganisms confer beneficial effects, promoting plant growth and enhancing health through direct and indirect mechanisms (Andreote et al. 2017; Aparicio et al. 2023).

Interactions between plants and microorganisms can be beneficial and symbiotic, where both parties share costs and benefits (Odum and Barret 2005). Mutualistic symbioses entail intimate and often obligatory relationships between microorganisms and specific host plants, typically requiring specialized structures for interaction, such as nodules in rhizobia-legume symbiosis and arbuscular mycorrhizas (Parniske 2008; Masson-Boivin et al. 2009). In contrast to mutualistic interactions, Plant Growth Promoting Rhizobacteria (PGPR) can interact with a wide range of plant species, showcasing broad taxonomic diversity, especially within Firmicutes and Proteobacteria phyla (Lugtenberg and Kamilova 2009; Drogue et al. 2012). Coined over 30 years ago, the term PGPR defines bacteria that must be nonpathogenic, coexist in the rhizosphere or colonize root surfaces, and have the ability to enhance plant yield through one or more mechanisms (Jha and Saraf 2015). For example, they can act through the solubilization of specific nutrients, the production of growth-promoting compounds (exudates), or the stimulation of the production of phytohormones (Hayat et al. 2010). Regarding nutrient supply through solubilization, bacterial activities that facilitate phosphate (and other minerals) solubilization are crucial. Many PGPR species, such as those from the genera *Pseudomonas*, *Bacillus*, or *Rhizobium*, can solubilize insoluble forms of phosphate by acidifying the external medium or by producing enzymes that hydrolyze phosphate-rich organic molecules (Richardson et al. 2009). For instance, the strain *Bacillus subtilis* GB03 emits volatile compounds capable of inducing the machinery required for iron acquisition in plants (Zhang et al. 2009; Romera et al. 2019).

In terms of exudate production, microorganisms play a crucial role in modulating the composition of plant exudates. The growth and composition of the root microbial environment are significantly influenced by the composition of these exudates, as many compounds within them attract bacteria, particularly those capable of metabolizing their components and thriving in that environment (Bais et al. 2006; Pothier et al. 2007; Badri et al. 2009; Drogue et al. 2012). On the other hand, the exudates can contribute to recruiting beneficial microorganisms while suppressing or inhibiting non-beneficial and pathogenic ones to prevent infections and the proliferation of harmful microbial communities (Zhang et al. 2009). These exudates also interact with organic and inorganic molecules in the soil, regulating their availability and absorption by the plant (Bertin et al. 2003). Additionally, certain plant responses, such as the stimulation of phytohormone production, can be modulated by ethylene. Ethylene, produced in response to various stressors such us extreme temperature, heavy metal presence, water scarcity, and also by the presence of microorganisms, can trigger responses that enhance plant survival in adverse conditions. Various microbial strains have been found to induce ACC synthase, catalyzing the production of ACC, an ethylene precursor, from S-adenosylmethionine (Glick 2012). It is noteworthy to observe the impact of certain microorganisms on specific plant species. For instance, inoculation of *Pachycereus pringlei* seedlings with the bacterium *Azospirillum brasiliense* not only promotes plant growth but also reduces the pH of the rhizosphere (Carrillo et al. 2002). The *P. simiae* WCS417 strain, initially isolated from wheat roots in the Netherlands in 1988, has been extensively studied for microbe–plant interactions (Pieterse et al. 2021). Research has predominantly focused on understanding how this PGPR induces plant defensive responses, particularly in *Arabidopsis thaliana* (Stringlis et al. 2018). This immune response, known as Induced Systemic Resistance (ISR), is triggered in *A. thaliana* by cellular components of the bacterium, such as flagellin (Yu et al. 2022). The WCS417 strain induces the *MYB72* gene, which encodes a transcription factor expressed under Fe-deficient conditions, thereby activating a response to Fe deficiency in *Arabidopsis* roots (Abedini et al. 2021). The WCS417 strain has been shown to promote growth in *Arabidopsis* plants, increasing the size of aboveground parts and chlorophyll levels, as well as enhancing root structure by promoting lateral root formation (Zamioudis et al. 2013). Other *Pseudomonas* strains have also been shown to promote growth in *A. thaliana* seedlings by producing pyoverdine, a siderophore synthesized under Fe-deficient conditions (Trapet et al. 2016). Under conditions of general nutrient deficiency in the soil and high pressure or CO_2_ levels, the WCS417 strain can promote plant growth by increasing fresh weight and leaf surface area (Williams et al. 2018). The growth-promoting effect could be attributed to auxins produced by certain microorganisms, with recent discoveries showing that this effect is dependent on auxin synthesis by the WCS417 strain (Zamioudis et al. 2014; Pieterse et al. 2021). However, there is no evidence that the growth-promoting effect of WCS417r depends on its ability to produce auxin. Separate findings indicate that WCS417 activates auxin-responsive pathways in *Arabidopsis thaliana*, can convert tryptophan into auxin, and promotes plant growth. However, there is no direct evidence linking these observations as a unified mechanism. Inoculation with the WCS417 strain or exposure to its volatiles can also induce genes related to Fe deficiency in *Arabidopsis* seedlings, such as the *IRT1* and *FRO2* genes (Zamioudis et al. 2015; Stringlis et al. 2018).

The objective of this article has been to determine the effect of inoculation with the WCS417 strain of *P. simiae* on the physiological responses to Fe deficiency in cucumber seedlings, on the gene expression codify these responses and to study the possible effect of this bacterial strain on promoting growth of cucumber plants under Fe deficiency conditions.

## Material and methods

### Bacteria strain, plant variety, and growth conditions

*P. simiae* WCS417 (resistant to rifampicin) was cultured at 27 °C in King´s B medium (20 g/L, 1.5 g/L K_2_HPO_4_, 1.5 g MgSO_4_, 15 mL/L glycerol, pH 7.2 ± 0.2) (King et al. 1954) supplemented with rifampicin (50 μg/mL). The cells were preserved in glycerol at -80 °C. Regarding the *Cucumis sativus* L*.* seeds used, this study employed cucumber seeds of the Ashley variety.

### Seed germination

Cucumber seeds were arranged on moistened blotting paper inside plastic trays. To expedite germination, 20 mL of 5 mM CaCl_2_ was added and they were covered with more moistened blotting paper. The tray was placed inside a plastic bag to prevent moisture loss and incubated for 2–3 days in a stove under dark conditions at 27 °C (sufficient time for the resulting seedlings to reach the appropriate size for subsequent cultivation in a hydroponic system).

They were grown in a growth chamber for 20–25 days at 22 °C (day) and 20 °C (night), with a relative humidity of 70% and a photoperiod of 14 h at an irradiance of 300 μmol m^−2^ s^−1^.

### Cultivation of bacteria and inoculum preparation

*P. simiae* WCS417 inoculum was obtained from a stock preserved in glycerol at −80 °C. They were cultured on KB agar plates (King’s medium B) supplemented with 50 μg/mL of rifampicin at 27 °C for 24 h. Subsequently, the cells were harvested using 10 mM MgSO_4_ after being washed twice by centrifugation for 5 min at 2400 g (FO685 Fixed-Angle Rotor). Finally, the cells were resuspended in 10 mM MgSO_4_, and the optical density (OD) at 600 nm was determined before inoculation.

### Experimental conditions

Once germinated and intentionally inducing slight etiolation, cucumber seedlings were transferred to the final hydroponic cultivation system. They were placed on plastic disks with a central hole where the seedling was positioned using a foam strip that wrapped around its etiolated stem, allowing the seedling to be anchored to the disk. Each of these disks, with its corresponding cucumber seedling, was placed in the holes of a polyethylene sheet arranged in a tray that floated on nutrient solution. This cultivation system ensured that the root was constantly in contact with the nutrient solution, which could be easily refreshed whenever necessary. Aeration system was installed to prevent anoxia situations. Römheld & Marchners nutrient solution was used (R&M) (Römheld and Marschner 1981) for hydroponic system, containing 2 mM Ca(NO_3_)_2_, 0.75 mM K_2_SO_4_, 0.65 mM MgSO_4_, 0.5 mM KH_2_PO_4_, 50 μM KCl, 10 μM H_3_BO_3_, 1 μM MnSO_4_, 0.5 μM CuSO_4_, 0.5 μM ZnSO_4_, 0.05 μM (NH_4_)_6_Mo_7_O_24_, and 10 μM Fe-EDDHA was used as the baseline Fe level during the days preceding the treatments application.

The cucumber plant treatments were exclusively conducted in hydroponic culture. Four treatments were applied: two control treatments; using the same nutrient solution described above, supplemented with 40 μM Fe-EDDHA control and Fe deficiency control (0 μM Fe-EDDHA), and two inoculated treatments; using the same nutrient solution described above, supplemented with 40 μM Fe-EDDHA inoculated and Fe deficiency inoculated (0 μM Fe-EDDHA). Each tray of plants, individually, received the corresponding treatment in a volume of 1.5 L of R&M nutrient solution. Regarding the inoculation, in all cases, the plants were inoculated with a concentration of 10^7^ colony-forming unit (CFU)/mL of bacteria directly in the hydroponic solution.

### Physiological determinations

#### Growth promotion

Periodic measurements of the height of all treatments' plants were taken. At the time of plant harvest, the fresh and dry weight of both the aboveground and root parts were determined.

#### Ferric reductase activity

Ferric reductase activity was determined as reflected in Lucena et al. (2006). To do this, intact plant roots underwent a washing pretreatment, involving immersion in a micronutrient-free nutrient solution at pH 5.5 for 30 min. Subsequently, they were transferred to the reducing capacity measurement solution, which contained 25 mL of micronutrient-free nutrient solution plus 100 mM Fe(III)-EDTA and 300 mM ferrozine at pH 5.0 (adjusted with 0.1 N KOH). The plants were incubated in this solution for 60 min under the same environmental conditions described earlier. The ferric reducing capacity was estimated using spectrophotometry. The presence of the Fe(II)–Ferrozine complex was quantified by determining absorbance at 562 nm, using an extinction coefficient of 29,800 M^−1^ cm^−1^ (Lucena et al. 2006). After the determination, the roots were separated from the plant, weighed, flash-frozen with liquid nitrogen, and stored at -80 °C for later use. The obtained results were expressed in relation to the fresh weight of the root and the time elapsed in the measurement solution.

#### Chlorophyll determination by SPAD levels

The level of chlorosis in the plants was determined through SPAD readings. A portable chlorophyll meter, Minolta SPAD-502, was employed. Three readings per leaf were taken, and the arithmetic mean of these readings was calculated. The readings were taken from the second pair of apical leaves. Young and expanded leaves.

#### Location of acidification and ferric reductase activity

For this determination, individual roots were dissected and sampled with the help of a scalpel, and they were kept in distilled water until later use. To verify the localization of the ferric reducing capacity, 100 mL of micronutrient-free nutrient solution at pH 5, gelled with 0.75% agar and supplemented with 300 mM ferrozine and 100 mM Fe(III)-EDTA, were poured onto a square Petri dish measuring 12 × 12 cm. Similarly, to analyze the localization of acidification, the same nutrient solution, this time with a pH adjusted to 6, gelled with 0.75% agar and added with 0.1% bromocresol purple acid/base indicator, was added to a Petri dish of similar characteristics to the one mentioned earlier. In both cases, before the solution completely gelled, the roots were placed on it and incubated in darkness for 30 min at room temperature. Once the gelation was complete and sufficient time had elapsed for both reactions (reduction and acidification) to manifest fully, photographs were taken.

#### qRT-PCR

The roots were crushed in a ceramic mortar to obtain a fine powder using liquid nitrogen. Total RNA was extracted using Tri Reagent solution (Molecular Research Center, Inc., Cincinnati, OH, USA) following the distributor's instructions and with the additional use of chloroform. After centrifugation, the RNA obtained in the pellet was precipitated using isopropanol. Several washes with 70% ethanol were performed to finally resuspend the obtained RNA by adding ultrapure water treated with diethylpyrocarbonate (DEPC). The concentration of the obtained RNA was quantified using spectrophotometry at 260 nm.

The M-MLV reverse transcriptase (Promega, Madison, WI, USA) was used to generate cDNA from 3 μg of RNA previously treated with DNase, using random sequence hexamers for amplification. The cDNA synthesis program used in the thermocycler (BIO-RAD T100) consisted of: 37 °C for 1 h, 42 °C for 30 min, 50 °C for 10 min, and 15 °C for 10 min.

The study of gene expression using RT-PCR was conducted using a thermocycler (qRT-PCR Bio-Rad CFX connect thermal cycler). The amplification profile consisted of 40 cycles with the following conditions: initial denaturation and polymerase activation (95 °C for 3 min), amplification and quantification (90 °C for 10 s, 57 °C for 15 s, and 72 °C for 30 s), and a final melting curve from 65 to 95 °C with an increment of 0.5 °C for 5 s to ensure the absence of primer dimers or nonspecific amplification products. PCR reactions were carried out in 20 μL of SYBR Green Bio-RAD PCR Master Mix following the distributor's instructions. To detect contamination in reaction components, negative controls with water were used. Normalization was performed using the combined action of two reference genes (*CsActin* and *CsCyclin*). The detailed primers used are listed in Table 1.

**Table 1:** Primer sequences used for qRT-PCR analysis in cucumber (*Cucumis sativus* L.)

Gene	Sequence (5´–3´)
*CsFRO1 (AY590765)*	F-ATACGGCCCTGTTTCCACTT
	R-GGGTTTTGTTGTGGTGGGAA
*CsIRT1 (AY590764)*	F-GCAGGTATCATTCTCGCCAC
	R-ATCATAGCAACGAAGCCCGA
*CsHA1 (AJ703810)*	F-GGGATGGGCTGGTGTAGTTTG
	R-TTCTTGGTCGTAAAGGCGGT
CsActin* (XM_004136807)	F-AACCCAAAGGCAAACAGGGA
	R-TCCGACCACTGGCATAGAGA
CsCyclo* (NM_001280769)	F-ATTTCCTATTTGCGTGTGTTGTT
	R-GTAGCATAAACCATGACCCATAATA

^*^Reference genes

### Statistical analysis

The statistical analysis and graphs were performed using GraphPad Prism 8. To compare data from the treatments, the Student's t test was used for parametric data, or the Mann–Whitney test for non-parametric data. For multiple comparisons, the ANOVA test was used for parametric data, or the Kruskal–Wallis test for non-parametric data. The significance level was determined by asterisks, with *P < 0.05, **P < 0.01, or ***P < 0.001 indicating the presence of significant differences between the treatments.

## Results

### Effect of strain WCS417 on growth promotion

**Fig. 1:**
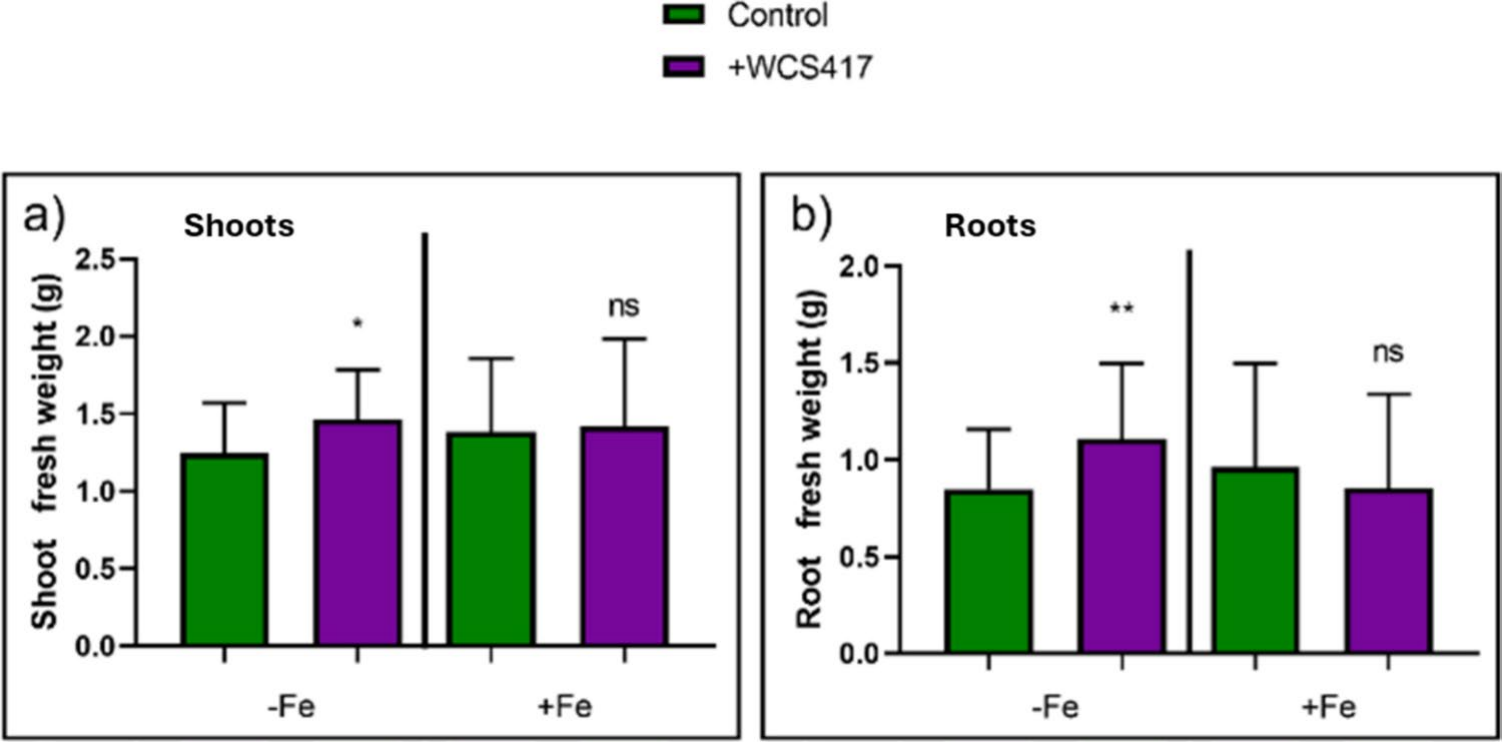
Effect of inoculation with WCS417 strain on shoots and roots weight of cucumber seedlings grown in nutrient solution with or without Fe. One week after the application of treatments, the plants were harvested. The root was excised from the aerial part and weighed separately. **a** Shoot fresh weight. **b** Root fresh weight. *P < 0.05 and **P < 0.01 indicate significant differences between inoculated and control treatments; ns, no significant differences. Values are the means ± SE of 30 replicates

Ten-day-old cucumber plants grown in hydroponic culture were subjected to treatments with and without Fe and were either inoculated or not with the WCS417 strain. Seven days after the application of treatments, the plants were collected, and the fresh weight of shoots and roots was separately determined. The results depicted in Fig. 1a, b demonstrate that inoculation promoted shoot and root growth in plants grown under Fe-deficient conditions.

### Effect of inoculation with the WCS417 strain on chlorophyll levels

**Fig. 2:**
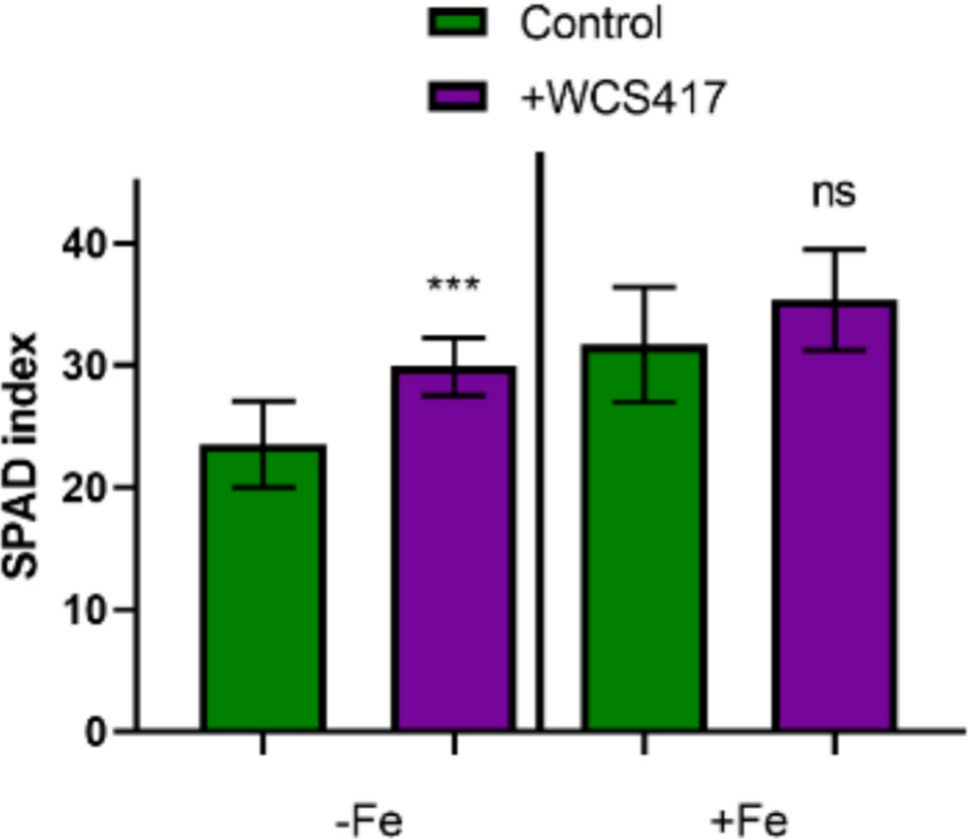
Effect of WCS417 strain on chlorophyll levels of cucumber seedlings grown in hydroponic culture with or without Fe. A SPAD meter was used on the youngest leaves of plants from the control and inoculated treatments. Both Fe-deficient (-Fe) and Fe-sufficient (+ Fe) treatments are shown. ***P < 0.001 indicates significant differences between inoculated and control treatments; ns, no significant differences. Values are the means ± SE of 6 replicates

A portable SPAD Minolta-502 m was used to take measurements on the apical leaves of cucumber plants after 4 days of treatment. The results indicated that under Fe-deficient conditions, cucumber plants inoculated with the WCS417 strain showed significantly higher levels of chlorophyll compared to non-inoculated plants (Fig. 2).

### Effect of inoculation on Fe reductase activity

**Fig. 3:**
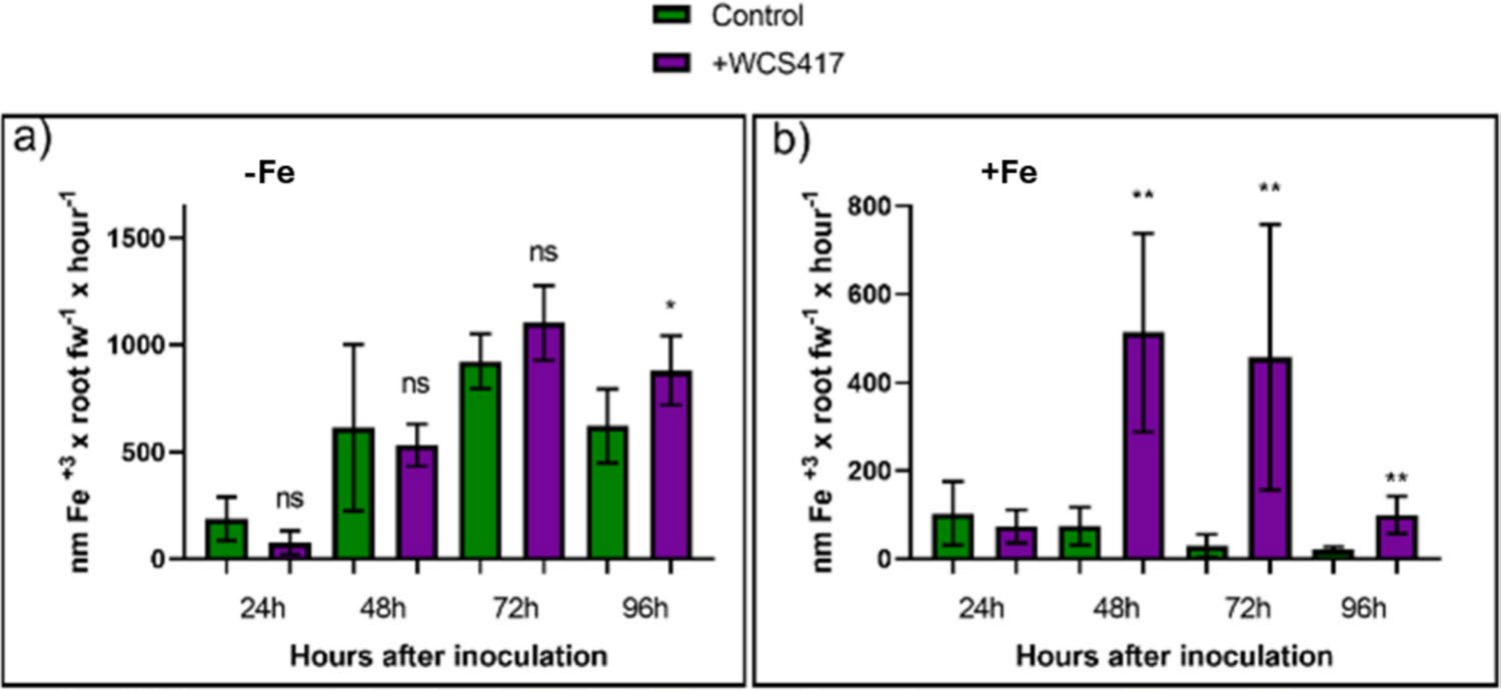
Effect of inoculation with WCS417 strain on ferric reductase activity of cucumber seedlings grown in nutrient solution with or without Fe. Ten-day-old seedlings were cultured in presence or absence of Fe. Half of the plants were inoculated with the WCS417 strain. Ferric reductase activity of the plants was determined every 24 h during a period of 4 days. **a** Ferric reductase activity under Fe-deficient conditions. **b** Ferric reductase activity under Fe-sufficient conditions. For each time, *P < 0.05 and **P < 0.01 indicate significant differences between inoculated and control treatments; ns, no significant differences. Values are the means ± SE of 6 replicates

Using the same growth conditions and treatments employed in the previous section, the role of the inoculation with the WCS417 strain on the ferric reductase activity of cucumber plants was determined. Under Fe-deficient conditions, significant differences exist only between the control and inoculated treatments at 96 h post-inoculation, with the inoculated treatment exhibiting higher ferric reductase activity (Fig. 3a). On the other hand, under Fe-sufficient conditions, at 48 h and 72 h post-inoculation, the inoculated treatment shows a substantial increase in ferric reductase activity compared to the control treatment. At 96 h, there is still a greater induction of ferric reductase activity in inoculated plants compared to control plants; however, this induction is much lower than that found in the preceding days (Fig. 3b). Additionally, as observed in the mentioned figure, there is a progressive decrease in activity over time from 48 to 96 h.

### Effect of strain WCS417 on rhizosphere acidification

**Fig. 4:**
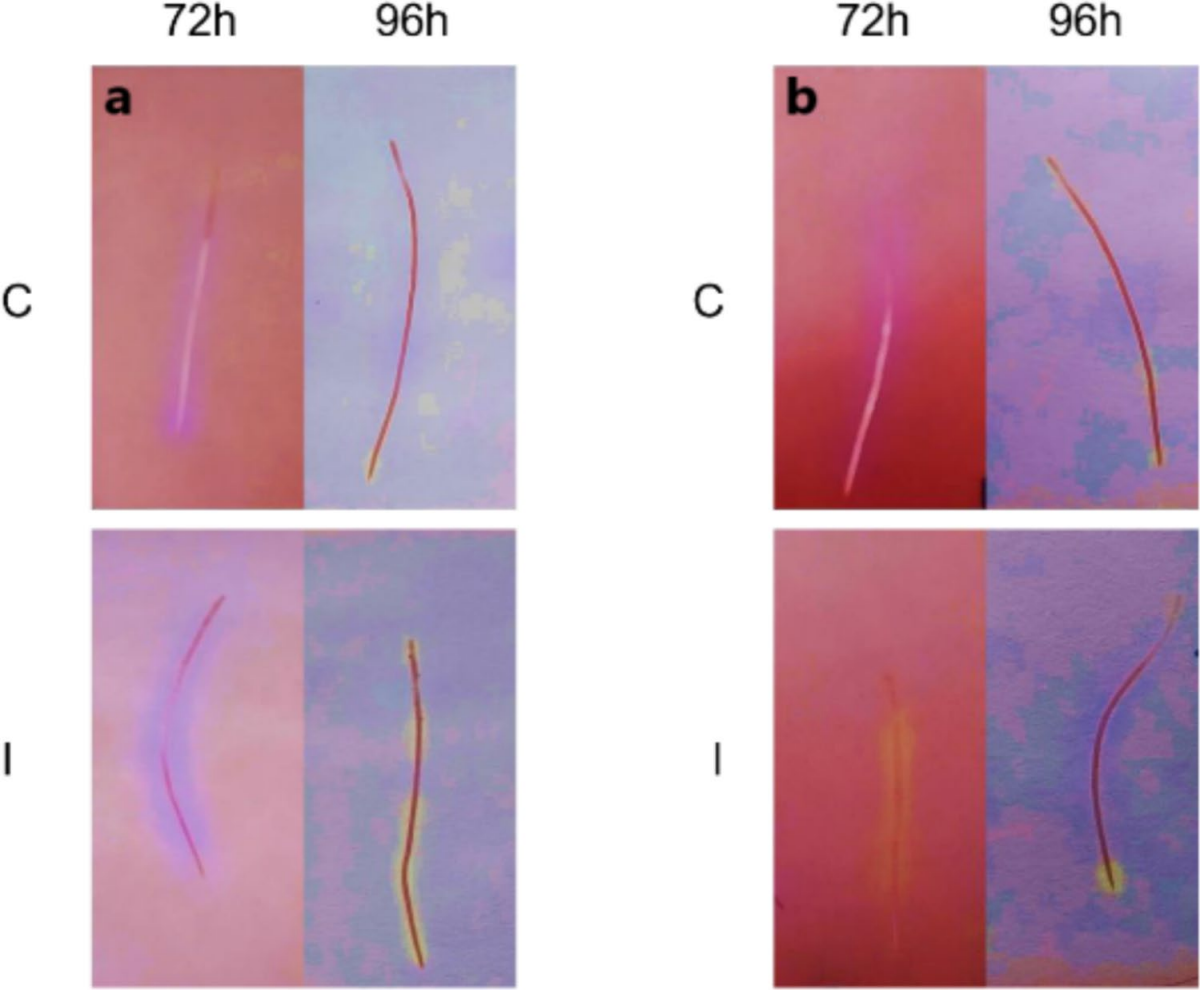
Effect of inoculation with WCS417 strain on rhizosphere acidification in roots of cucumber seedlings grown in hydroponic culture with or without Fe. After treatments, whole root samples were taken every 24 h for 4 days and placed on an agar plate with an acid–base indicator (bromocresol purple). The roots collected at 72 h and 96 h are shown in the figure. **a** Fe deficiency. **b** Fe sufficiency. C, Control; I, inoculated

After 24 and 48 h from the application of treatments, no significant response was observed under both Fe-sufficient and Fe-deficient conditions (results not shown). At 72 h post-inoculation, the roots of inoculated plants grown under Fe sufficiency showed a greater acidification response than the control treatment seedlings (Fig. 4b). At 96 h post-inoculation under Fe-deficient conditions, although acidification occurred in both the control and inoculated treatments, this effect was much more pronounced in the inoculated plants (Fig. 4a). Under Fe sufficiency conditions (Fig. 4b), at 96 h, a higher acidification effect was also observed in the roots of seedlings inoculated with the WCS417 strain.

### Effect of inoculation with the WCS417 strain on the expression levels of the genes involved in Fe deficiency responses

**Fig. 5:**
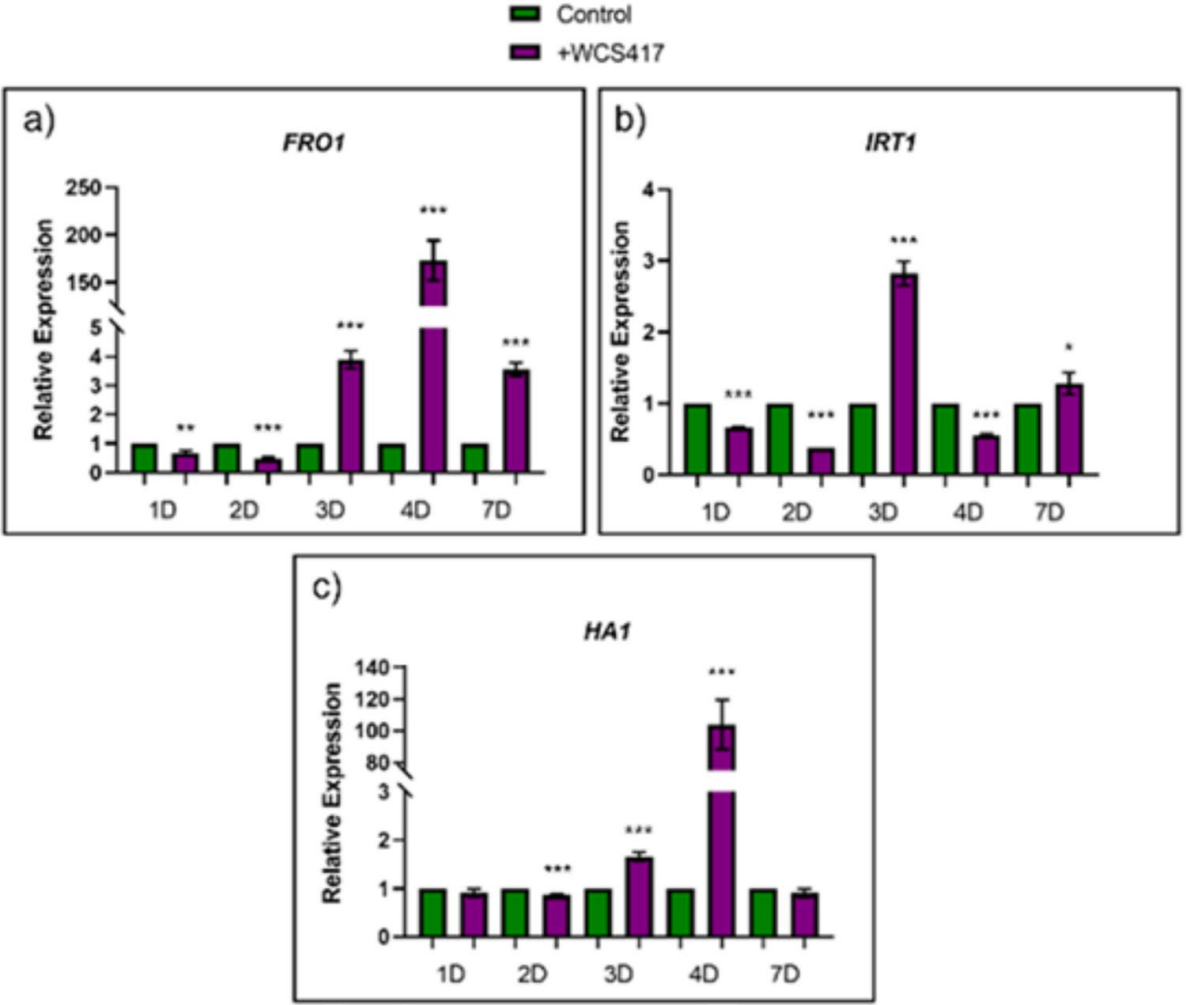
Effect of inoculation with WCS417 strain on gene expression of Fe-related genes of cucumber plants grown under Fe sufficiency. Ten-day-old cucumber seedlings grown in hydroponic culture were cultured in presence of 40 μM Fe-EDDHA. Half of the seedlings were inoculated with WCS417 strain. Samples were taken during 7 days. Total RNA was retrotranscribed to cDNA to determine gene expression by RT-PCR, relative expression was calculated in relation to the control of each day. **a** Ferric reductase, *FRO1*. **b** Fe^2+^ transporter. **c** H^+^-ATPase. Bars represent the mean ± SE of two biological repeats and three technical repeats. Within each time, *P < 0.05, **P < 0.01 or ***P < 0.001 indicate significant differences in relation to the control treatment

*FRO1*, *IRT1*, and *HA1* are genes that codify ferric reductase enzyme, Fe^2+^ transporter protein, and H^+^-ATPase, respectively. It was confirmed that inoculation with the WCS417 strain in cucumber seedlings grown under Fe sufficiency promoted induction of the *FRO1* gene at 3, 4, and 7 days post-inoculation, with this effect being particularly notable at 3 days post-inoculation. However, during the first and second days post-inoculation, there was an inhibition of the gene (Fig. 5a). Regarding the gene encoding the Fe^2+^ transporter, *IRT1*, induction was observed at 3 and 7 days post-inoculation, with a very mild induction at 7 days. Similarly, there was an inhibition of the gene on the first and second days post-inoculation (Fig. 5b). As for the gene encoding H^+^-ATPase, *HA1*, a strong induction occurred at 4 days due to inoculation and a mild induction at 3 days (Fig. 5c).

## Discussion

*P. simiae* strain WCS417 is one of the best characterized PGPR bacteria. It is capable of colonizing the external surface of the root of plants such as, for example, *Arabidopsis thaliana*, although it also has the ability to endophytically colonize tomato plants (Pieterse et al. 2021). There is evidence suggesting that the growth-promoting effect and changes in root architecture provoked by this strain are due to production of auxin (Ashraf et al. 2013). Likewise, its growth promotion has been attributed to other mechanisms such as, for example, inhibition of the growth of other microorganisms (Duijff et al. 1993).

It has been shown that root inoculation of dicotyledonous plants with the WCS417 strain triggers the induction of ISR in distal tissues, protecting against a wide range of pathogens and herbivores (Pieterse et al. 2021). It is known that the WCS417 strain is capable of inducing the *MYB72* gene, which encodes the homonymous transcription factor involved in both ISR and the response to Fe deficiency (Zamioudis et al. 2014). Along these lines, it has been shown that the volatiles produced by the WCS417 strain are capable of promoting the induction of genes involved in Fe deficiency in *A. thaliana* seedlings (Zamioudis et al. 2015).

One objective of this work has been to test the effect of the *P. simiae* strain WCS417 on the response mechanisms to Fe deficiency in plants with agronomic interest such as cucumber. The results obtained have shown the growth-promoting effect of the WCS417 strain on cucumber plants grown under Fe deficiency (Fig. 1). Although, in this case, the cucumber plants grown under sufficient Fe conditions did not respond positively to the treatment with WCS417, our research group have recently been able to demonstrate that the application of the WCS417 induces the growth of tomato plants grown in hydroponic culture under sufficient Fe conditions (40 μM Fe-EDDHA) (Aparicio et al. 2025). Other authors had already highlighted this growth-promoting effect (Verbon et al. 2019). Along these same lines, the inoculation of *Vigna radiata* plants with the GRP3A strain of *Pseudomonas* produced a clear growth-promoting effect both under Fe sufficiency and deficiency. In addition, this strain is capable of producing siderophores in conditions of absence of Fe, which help the plant to alleviate the deficiency conditions of this element (Sharma and Johri 2003). Similarly, Ali et al. (2011) demonstrated that the inoculation of wheat plants with the AKMP7 strain of *Pseudomonas putida* promoted the growth of both the root and the aerial part (through length and dry weight determinations). In a similar way, Sandhya et al. (2010) demonstrated that five different strains of the *Pseudomonas* genus were capable of inducing growth promotion in cultivated corn plants under water stress conditions, demonstrating the agronomic potential of members of this genus in different environmental stress situations. In relation to alkaline pH, it is known that growth-promoting microorganisms, especially alkaliphiles, can promote plant resistance to these conditions through the production of IAA (auxins), ACC, and by increasing internal K levels. In this way, the relative humidity of the plant and its ionic homeostasis are maintained (Msimbira and Smith 2020). Ipek et al. (2014) used five different strains with a growth-promoting effect on strawberry plants grown in limestone soil, and a highly significant growth-promoting effect was produced with all strains.

With respect to chlorosis, although under Fe sufficiency, there was no difference between control and inoculated treatment, under Fe deficiency, cucumber plants inoculated with the WCS417 strain showed much higher levels of SPAD than control plants (Fig. 2). These results seem to indicate that the WCS417 strain can produce plant greener under Fe deficiency. Rahimi et al. (2020) inoculated quince plants (*Cydonia oblonga*) with *P. fluorescens* and *M. yunnanensis*. Similarly, inoculated plants grown under Fe deficiency showed much higher levels of chlorophyll than control plants. Verbon et al. (2019) inoculated *A. thaliana* seedlings with the WCS417 strain both under Fe sufficiency and deficiency. However, in that occasion, only the plants inoculated and grown under Fe sufficiency reached a pronounced increase in chlorophyll levels in a period of 7 days. These differences may be due, at least in part, to the experimental designs used for each species. In the case of *A. thaliana*, seeds were sown at low density on standard growth medium, with a final concentration of 40 µM or 200 µM FeNaEDTA, in square Petri dishes measuring 120 × 120 mm. Seedlings aged between 14 and 19 days were used for chlorophyll quantification, with dimethylsulfoxide (DMSO) employed for pigment extraction. By contrast, in our experiments, cucumber seedlings were grown in hydroponic culture with or without iron supplementation (40 µM Fe-EDDHA). A SPAD meter was used to assess chlorophyll levels in the youngest leaves of 24 day old cucumber plants from both control and inoculated treatments.

Inoculation of cucumber plants with the WCS417 strain produced a highly significant increase in ferric reductase activity at 48, 72, and 96 h after inoculation under Fe sufficiency (Fig. 3b). Under Fe deficiency, there was only an increase in this activity at 96 h (Fig. 3a). In a similar way, Rahimi et al. (2020) obtained a very high increase in ferric reductase activity due to the inoculation of quince plants with *Pseudomonas fluorescens* and *Microccucuce yunnanensis* under Fe deficiency. Although there was a slight increase in ferric reductase activity in inoculated plants under Fe deficiency, this was not as pronounced as what occurred under Fe sufficiency. Following this same line, Martínez-Medina et al. (2017) demonstrated that *A. thaliana* seedlings grown under Fe sufficiency and exposed to the volatiles generated by *Trichoderma* manifested an induction of ferric reductase activity. Zhou et al. (2016) inoculated *A. thaliana* seedlings grown at various concentrations of Fe with the BFKC01 strain of *Paenibacillus polymyxa*. At the highest concentrations, that is, under Fe sufficiency, the seedlings showed an induction of ferric reductase activity due to the effect of the inoculation.

Inoculation of cucumber plants with the WCS417 strain caused a much more intense acidification response than in control plants under both Fe sufficiency and Fe deficiency (Fig. 4). Similarly, our research group have got results relative to inoculations by direct application to the nutrient solution or by foliar spraying of tomato plants grown in the presence of bicarbonate (Aparicio et al. 2025). In both cases, it produced a quite evident acidification effect of the medium. The fact that the response to acidification occurs both when the plant is inoculated in nutrient solution and by foliar spray shows that this response is produced by plant mechanisms, possibly through the extrusion of protons through the root. Carrillo et al. (2002) demonstrated that the inoculation of giant cactus plants *Pachycereus pringlei* with *Azospirillum brasilense* in the presence of ammonium induced the acidification response of the rhizosphere in the subapical zone of the roots after 72 h. Another example of acidification due to the influence of a PGPR was provided by Zhang et al. (2009). After exposure of *A. thaliana* seedlings to volatiles produced by the GB03 strain of *Bacillus subtilis*, there was a strong induction of the rhizosphere acidification response.

Inoculation with the WCS417 strain also affected the expression of genes involved in the Fe deficiency response. Under Fe sufficiency, a strong induction of these genes was detected at certain times, especially the *FRO* and *HA1* genes (Fig. 5). In a similar way, exposure of *A. thaliana* seedlings to volatiles produced by the WCS417 strain resulted in a strong induction of the *IRT1* and *FRO* genes, especially after 3 days (Zamioudis et al. 2015). Martínez-Medina et al. (2017) also detected a strong induction of genes involved in Fe deficiency responses in *A. thaliana* and tomato plants, such as *FRO* or *IRT1*, after 2 days due to the effect of exposure to volatiles from two strains of *Trichoderma sp.* The fact that inoculation with the WCS417 strain induces the expression of genes related to iron-deficiency response mechanisms in cucumber plants grown under sufficient Fe conditions reinforces the hypothesis that this strain acts at the molecular level regardless of Fe availability in the medium, making it a strong candidate to become a biofertilizer capable of correcting iron chlorosis. Recently, Aparicio et al. (2025) have shown the ability of the *P. simiae* WCS417 strain to induce medium acidification in the presence of bicarbonate to increase the SPAD index and to improve the growth and development of the tomato plants in calcareous conditions provoked by the presence of bicarbonate. In conclusion, our results shown that inoculation of cucumber plants with *P. simiae* strain WCS417 promotes shoot and root growth in absence of Fe. It also enhances ferric reductase activity of cucumber plants cultured in presence of Fe after 48 h and chlorophyll levels of cucumber plants cultured in the absence of Fe. Furthermore, WCS417 strain strongly increases relative expression of Fe-related genes at certain times in the presence of Fe and induces acidification response in cucumber plants cultured in presence or absence of Fe. Although further research is required to clarify the mode of action, our results underscore the potential of *P. simiae* WCS417 as an effective biofertilizer for a sustainable Fe nutrition of dicot plants.

## Data Availability

The original contributions presented in the study are included in the article. Further inquiries can be directed to the corresponding author.

## Abbreviations

ACC: 1-Aminocyclopropane-1-carboxylic acid
*FRO1*: Ferric reductase oxidase 1
*HA1*: H^+^-ATPase gene
*IRT1*: Iron-regulated transporter 1
PGPR: Plant growth-promoting rhizobacteria
SPAD: Soil plant analysis development meter
WCS417: *Pseudomonas **simiae* Strain WCS417
